# Association Between Cardiovascular Risk Assessed by the SCORE System and Cardiac Computed Tomography-Derived Left Atrioventricular Coupling Index

**DOI:** 10.3390/diagnostics15091075

**Published:** 2025-04-24

**Authors:** Przemysław Cheładze, Michał Fułek, Katarzyna Fułek, Rafał Poręba, Paweł Gać

**Affiliations:** 1 Centre for Diagnostic Imaging, 4th Military Hospital, 50-981 Wroclaw, Poland; 2 Department of Diabetology, Hypertension and Internal Diseases, Institute of Internal Diseases, Wroclaw Medical University, 50-556 Wroclaw, Poland; 3 Department of Otolaryngology, Head and Neck Surgery, Wroclaw Medical University, 50-556 Wroclaw, Poland; 4 Department of Biological Principles of Physical Activity, Wroclaw University of Health and Sport Sciences, 51-612 Wroclaw, Poland; 5 Department of Environmental Health, Occupational Medicine and Epidemiology, Wroclaw Medical University, 50-345 Wroclaw, Poland

**Keywords:** left atrioventricular coupling index, cardiac computed tomography, cardiovascular risk, SCORE

## Abstract

**Background/Objectives**: Recent advancements in cardiovascular imaging have opened new avenues for integrating novel biomarkers into risk assessment models, enhancing their predictive accuracy. One such emerging biomarker is the left atrioventricular coupling index (LACI). The study aims to evaluate the relationship between the SCORE (Systematic Coronary Risk Evaluation) and LACI derived from cardiac computed tomography (CCT). **Methods**: This study included 137 participants (56.09 ± 7.64 years). Cardiovascular risk was assessed using the SCORE system. CCT was performed using the standard coronary computed tomography angiography protocol. LACI was calculated as the ratio of left atrial end-diastolic volume (LA EDV) to left ventricular end-diastolic volume (LV EDV), expressed as a percentage. **Results**: The subgroup with SCORE ≥5% had higher LACI than the subgroup with SCORE < 5%. Similarly, the subgroup with SCORE ≥10% had higher LACI than the subgroup with SCORE < 10%. LACI demonstrated a significant positive correlation with the SCORE (r = 0.29, *p* = 0.01). Prediction analysis showed that LACI ≥ 53.34% as a predictor of SCORE ≥ 10% had the highest accuracy of 78.1%, with a high sensitivity of 79.8% and a moderate specificity of 61.5%. High specificity (80.6%) is characterized by LACI ≥ 29.52% as a predictor of SCORE ≥ 5. **Conclusions**: LACI is a novel and significant biomarker associated with cardiovascular risk, as reflected by its relationship with the SCORE system.

## 1. Introduction

Cardiovascular disease (CVD) remains the leading cause of morbidity and mortality worldwide [1]. Despite significant advancements in prevention and treatment, the global burden of CVD continues to rise [2,3,4], largely driven by modifiable risk factors such as hypertension [5], dyslipidemia [6], smoking [7], metabolic dysfunction-associated steatotic liver disease and diabetes (MASLD) [8], obesity [9], sleep disorders [10] as well as non-modifiable factors like age and genetic predisposition [3]. Accurate risk stratification, which refers to the assessment of an individual’s likelihood of experiencing cardiovascular events such as myocardial infarction or stroke within a specified timeframe, is therefore crucial for effective prevention and intervention strategies.

Traditional risk assessment tools, including the SCORE (Systematic Coronary Risk Evaluation) [11], Framingham Risk Score, QRISK3, Reynolds Risk Score, and ASCVD Risk Calculator, rely heavily on clinical and laboratory parameters to estimate cardiovascular risk. These tools have significantly contributed to identifying high-risk individuals in the general population and guiding therapeutic decisions. However, they often face limitations in providing tailored risk assessments, particularly in intermediate-risk groups, due to their reliance on general population data and static variables. Furthermore, their applicability across diverse populations with varying baseline risk profiles remains a challenge.

Recent advancements in cardiovascular imaging have opened new avenues for integrating novel biomarkers into risk assessment models, enhancing their predictive accuracy [12,13]. One such emerging biomarker is the left atrioventricular coupling index (LACI), derived from cardiac computed tomography (CCT) [14]. LACI reflects the relationship between left atrial and left ventricular end-diastolic volumes, offering insights into cardiac remodeling and hemodynamic stress that may not be captured by traditional risk factors [15]. By incorporating structural and functional dimensions of cardiac anatomy, LACI has the potential to bridge the gap between conventional clinical parameters and individualized risk stratification.

LACI is calculated as the ratio of left atrial end-diastolic volume (LA EDV) to left ventricular end-diastolic volume (LV EDV), expressed as a percentage. This parameter provides a composite measure of the interplay between atrial and ventricular mechanics, which plays a critical role in maintaining optimal cardiac output and preventing adverse remodeling.

## 2. Objective

The objective of this study is to evaluate whether the LACI, derived from CCT, is associated with cardiovascular risk as assessed by the SCORE (Systematic Coronary Risk Evaluation) system. Specifically, this study aims to explore its potential as a structural and functional marker reflecting cardiovascular burden in individuals with varying SCORE-defined risk levels.

## 3. Materials and Methods

The study entitled “Selected quantitative parameters of coronary computed tomography angiography as markers of cardiovascular health” was conducted in the computed tomography laboratory of the 4th Military Hospital in Wroclaw as part of the project of the Wroclaw Medical University entitled “The importance of selected methods of laboratory, imaging and electrophysiological diagnostics in the assessment of cardiovascular health”. Patients were recruited for the project between January 2023 and December 2023.

The inclusion criteria for the study were age ≥ 40 and clinical indication to CCTA according to the recommendations of the European Society of Cardiology [16]. The exclusion criteria were coronary artery calcium score value exceeding 1500, insufficient quality of the coronary computed tomography angiography, previous coronary interventions, previous myocardial infarction, previous stroke, heart failure, diabetes, chronic kidney disease (based on KDIGO 2012 definition), and hyperthyroidism or hypothyroidism.

Group size was determined using a sample size calculator. The selection conditions were as follows: population size 3.0 million, fraction size 0.5, maximum error 10%, confidence level 95%. The required minimum size of the study group was 96. This study included a total of 137 participants, with a mean age of 56.09 ± 7.64 years, of whom 54% were female. This size of the study group determines the maximum study error at the level of 8%. Among the patients recruited to the study, clinical indications for CCTA were CAD suspicion (84 patients, 61.3%), chest pain (49, 35.8%), low intermediate CAD risk (42, 30.6%), numerous CAD risk factors (40, 29.2%), inconclusive or non-diagnostic exercise test (21, 15.3%), and sudden cardiac death in the family history (1, 0.7%). Detailed baseline characteristics of the study population are presented in Table 1.

All participants provided informed consent prior to inclusion, and the study protocol was approved by the Ethics Committee of Wroclaw Medical University (ID: KB-210/2023).

Data on anthropometric measurements, smoking status, and blood pressure (BP) were collected during routine clinical evaluations. Laboratory analyses included measurements of total cholesterol and triglyceride levels, which were performed using standard enzymatic methods.

Cardiovascular risk was assessed using the SCORE (Systematic Coronary Risk Evaluation) system designed for the Polish population. SCORE estimates the risk of fatal cardiovascular events within 10 years of assessment. SCORE was estimated based on age, sex, tobacco intake, systolic blood pressure, and blood total cholesterol concentration. Based on SCORE, percentage risk of death within 10 years caused by cardiovascular incidents was assumed as small (when SCORE < 1%), moderate (when SCORE 1–4%), increased (when SCORE 5–9%), or significantly increased (when SCORE ≥ 10%).

Transthoracic echocardiography was performed according to a standard protocol. The dimensions of the cardiac chambers, as well as systolic and diastolic function of the left ventricle, were analyzed. The following parameters of cardiac morphology were assessed: left ventricular end-diastolic dimension (LVEDD) and end-systolic dimension (LVESD), interventricular septal thickness in diastole (IVSEDD), posterior wall thickness in diastole (PWEDD), and left atrial dimension (LA). Left ventricular systolic function was determined by measuring the left ventricular ejection fraction (LVEF) using the Simpson method. Left ventricular diastolic function was assessed using pulsed-wave Doppler and tissue Doppler. Pulsed-wave Doppler was used to measure the ratio of the maximum early diastolic mitral inflow velocity (E) to the maximum late diastolic mitral inflow velocity (A), i.e., E/A ratio. Tissue Doppler was used to measure the early diastolic mitral annular velocity (E’). The E/E’ ratio was determined.

**Figure 1:**
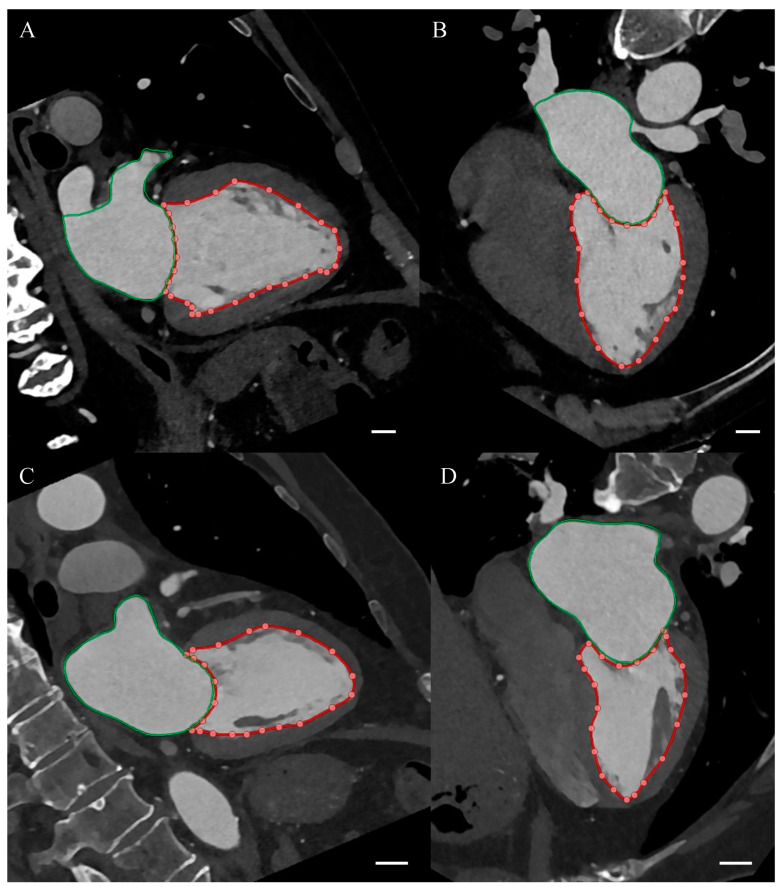
Cardiac computed tomography. The red curve with dots indicates the outline of the left ventricle, the green curve indicates the outline of the left atrium. The white section in the lower right corner of each panel is 1 cm long on the image magnification scale. (**A**) Two-chamber view in a patient with a left atrioventricular coupling index (LACI) of 21%. (**B**) Four-chamber view in a patient with LACI of 21%. (**C**) Two-chamber view in a patient with LACI of 56%. (**D**) Four-chamber view in a patient with LACI of 56%.

Cardiac computed tomography (CCT) was performed using the standard coronary computed tomography angiography (CCTA) protocol with dual source 384-slice CT scanner SOMATOM Force (Siemens Healthcare, Erlangen, Germany). The obtained images were assessed by a certified radiologist with EACVI Cardiac Computed Tomography Exam and over 10 years of clinical experience. The coronary artery calcium score (CACS) was assessed on native phase images of CCTA examination. The coronary artery disease severity was determined based on the Coronary Artery Disease—Reporting and Data System (CAD-RADS), where 0—documented absence of coronary artery disease (CAD), 1—minimal non-obstructive CAD (maximal stenosis: 1–24%), 2—mild non-obstructive CAD (maximal stenosis: 25–49%), 3—moderate CAD (maximal stenosis: 50–69%), 4—severe CAD (maximal stenosis: 70–99%), and 5—total coronary artery occlusion. Left atrioventricular coupling index (LACI) was calculated as the ratio of left atrial end-diastolic volume (LA EDV) to left ventricular end-diastolic volume (LV EDV), expressed as a percentage. LA EDV and LV EDV were assessed semi-automatically using the CT Cardiac Functional Analysis post-processing application (Siemens Healthcare, Erlangen, Germany). Example CCT images from a patient with LACI of 21% and 56% are presented in Figure 1.

Participants were stratified into subgroups based on variables included in the SCORE assessment, SCORE categories CAD-RADS ≥ 3, and LACI median values.

The statistical evaluations were conducted using “Dell Statistica 13.1” software (Dell Inc., Round Rock, TX, USA). Quantitative data were presented in the following format: mean ± standard deviations. Depending on the normality of the distribution tested by the Shapiro–Wilk test, parametric tests (for variables with a normal distribution) and nonparametric tests (for variables with a non-normal distribution) were used to test hypotheses. In the comparative analysis of 2 means, the *t* test or the Mann–Whitney U test were used, respectively. Qualitative data were represented as percentages. For qualitative variables in comparative analyses, the chi-square test was used. To determine the relationship between the studied variables, correlation analysis was performed. In the case of quantitative variables with a non-normal distribution, Pearson’s correlation coefficients were determined; in the case of quantitative variables with a non-normal distribution—Spearman’s coefficients. Prediction analysis was performed using ROC curves and analysis of test sensitivity and specificity. Statistical significance was attributed to results with a *p*-value of less than 0.05.

## 4. Results

The distribution of participants based on SCORE categories is summarized in Table 1. Among the cohort, 12.4% had a SCORE < 1%, 65.0% fell within the 1–4% range, 13.1% had a SCORE between 5 and 9%, and 9.5% were classified as having a SCORE ≥ 10%.

The results of echocardiography and computed tomography angiography of the coronary arteries are presented in Table 2. On echocardiography, LA was 41.46 ± 4.03 mm, LVEDD 50.44 ± 5.11 mm, and LVEF 65.70 ± 5.72%. In CCTA, 43.8% had no coronary artery disease, 43.8% had insignificant coronary artery stenoses, 9.5% had moderate coronary artery stenoses, and 2.9% had significant coronary artery stenoses or occlusions of coronary arteries. The mean left atrioventricular coupling index (LACI) was 42.56 ± 21.73%.

Statistically significant differences in LACI were observed between sex subgroups, with higher values recorded in males compared to females. Similarly, participants with systolic blood pressure (BP) ≥ 140 mmHg had significantly elevated LACI values compared to those with BP < 140 mmHg. The subgroup with SCORE ≥ 5% had higher LACI than the subgroup with SCORE < 5%. Similarly, the subgroup with SCORE ≥ 10% had higher LACI than the subgroup with SCORE < 10%. LACI was also significantly higher in patients with at least moderate coronary artery stenoses (CAD-RADS ≥ 3) than in patients without significant coronary artery stenoses (CAD-RADS < 3). Left atrioventricular coupling index (LACI) in subgroups determined based on the SCORE questionnaire is presented in Table 3.

Furthermore, the subgroup with LACI ≥ median (≥31.84%) had higher SCORE than the subgroup with LACI < median (<31.84%). SCORE in subgroups determined based on the left atrioventricular coupling index (LACI) is presented in Table 4.

**Figure 2:**
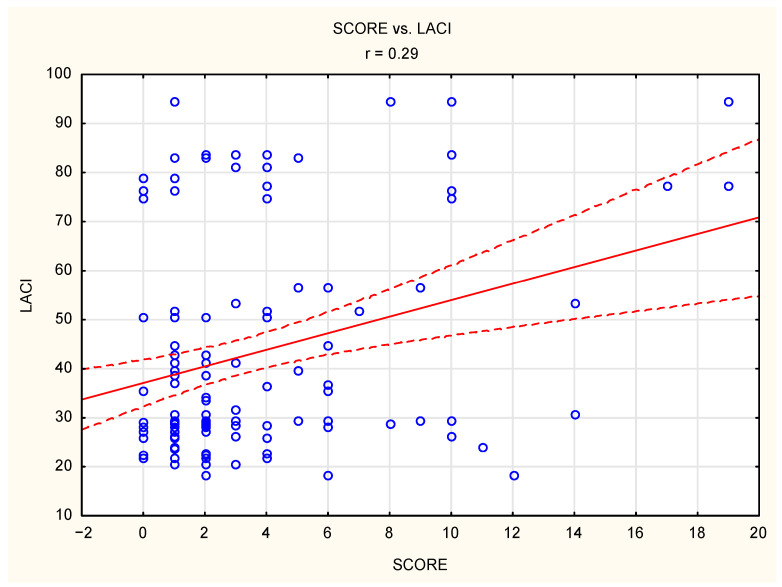

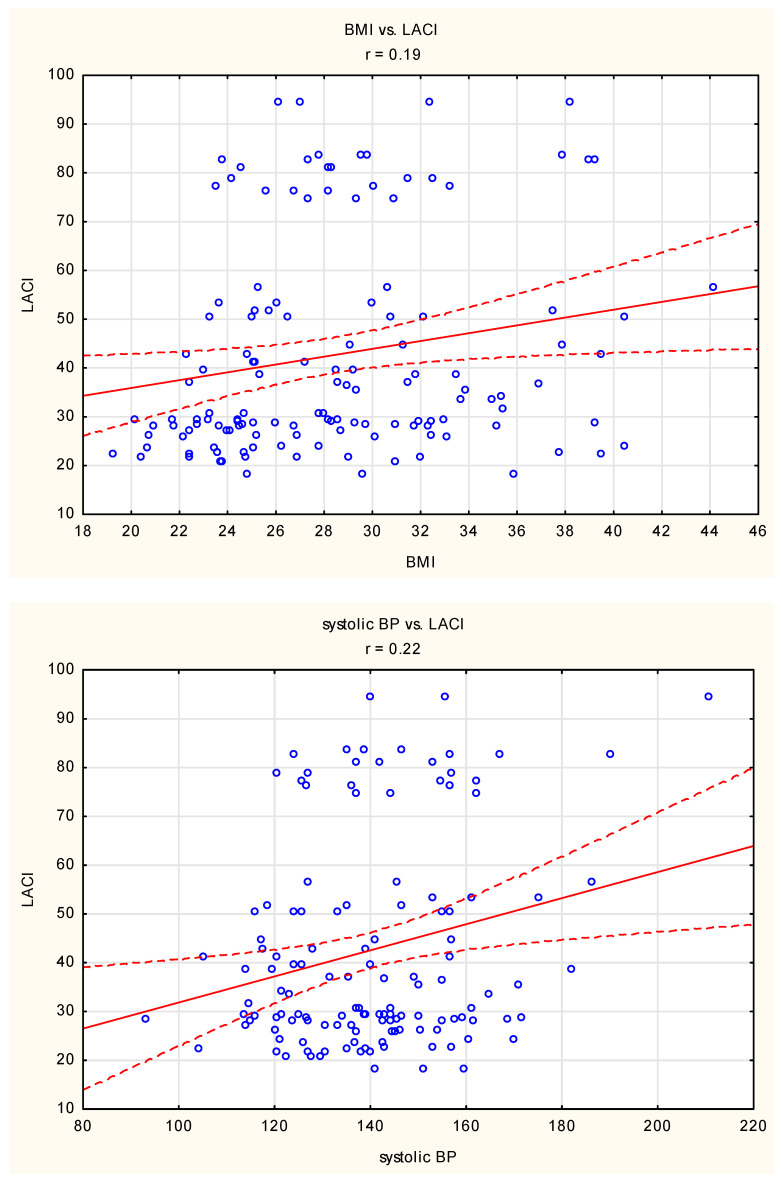

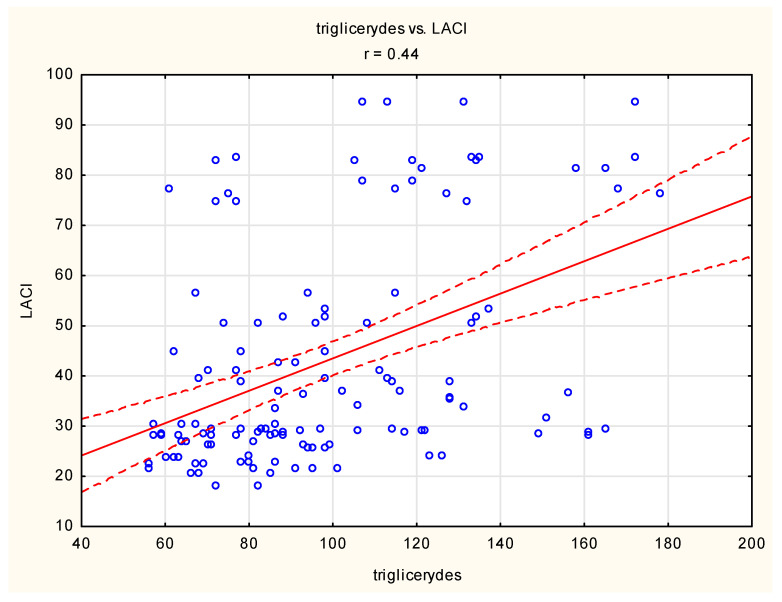
Significant correlations in the studied group of patients.

LACI demonstrated a significant positive correlation with the SCORE (r = 0.29, *p* = 0.01), indicating its potential as a marker of overall cardiovascular risk. Additional significant correlations were observed with body mass index (BMI; r = 0.19, *p* = 0.03), systolic BP (r = 0.22, *p* = 0.01), and triglyceride levels (r = 0.44, *p* = 0.01). The results of the correlation analysis are presented in Table 5, and statistically significant correlations are presented in Figure 2.

The predictive performance of LACI for cardiovascular risk stratification was assessed using receiver operating characteristic (ROC) curves. Prediction analysis showed that LACI ≥ 53.34% as a predictor of SCORE ≥ 10% had the highest accuracy of 78.1%, with a high sensitivity of 79.8% and moderate specificity of 61.5%. High specificity (80.6%) is characterized by LACI ≥ 29.52% as a predictor of SCORE ≥ 5%. The results of the analysis of the predictive value of LACI in relation to SCORE risk are presented in Table 6.

**Figure 3:**
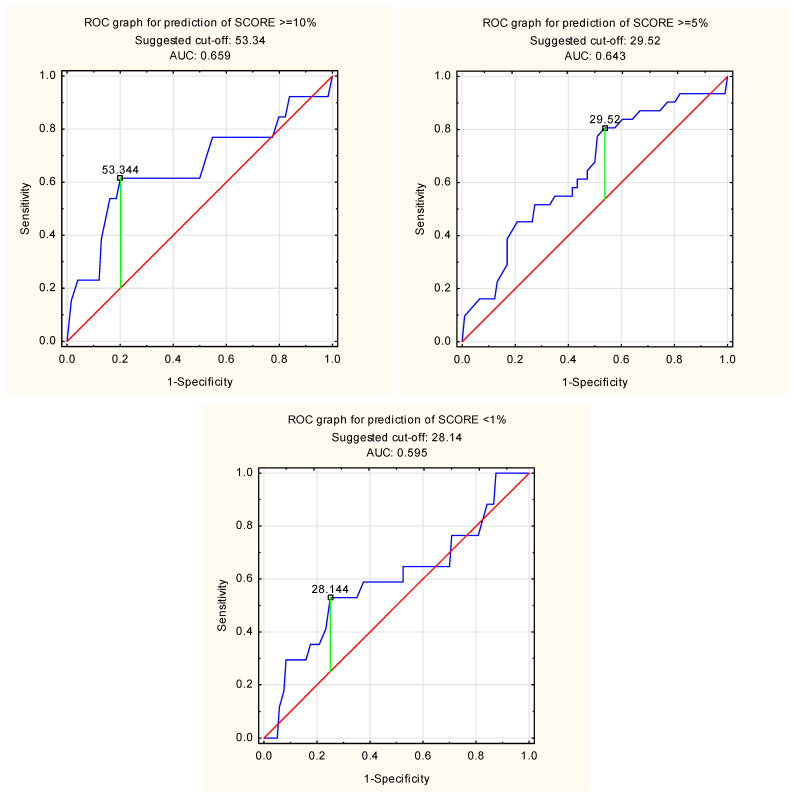
ROC curve for prediction of SCORE ≥ 10%, SCORE ≥ 5%, and SCORE < 1% using LACI.

Figure 3 shows the ROC curve for predicting SCORE ≥ 10%, SCORE ≥ 5%, and SCORE < 1% using LACI.

## 5. Discussion

The findings of this study indicate that the left atrioventricular coupling index (LACI) shows a statistically significant association with cardiovascular risk as assessed by the SCORE system. Participants with higher LACI values tended to fall into higher SCORE categories, suggesting that LACI may reflect certain structural or hemodynamic changes related to overall cardiovascular burden.

Moreover, this study identified key demographic and clinical factors that influence LACI values, including sex, systolic blood pressure (BP), and body mass index (BMI). Males exhibited higher LACI values compared to females, and participants with systolic BP ≥140 mmHg showed significantly elevated LACI levels. These findings underscore the multifactorial nature of LACI and its responsiveness to both hemodynamic and metabolic parameters.

This study builds upon prior research linking alterations in atrial and ventricular volumes with adverse cardiovascular outcomes, such as heart failure and arrhythmias. Enlarged left atrial volume, for instance, has been associated with atrial fibrillation and poor cardiovascular prognosis [17,18], while increased left ventricular end-diastolic volume has been linked to heart failure with reduced ejection fraction (HFrEF) [19]. By integrating these dimensions, LACI provides a comprehensive measure of atrial–ventricular interactions, offering a novel perspective on cardiovascular risk.

Recent evidence supports the utility of LACI in clinical practice [20,21]. Increasingly, studies demonstrate the predictive value of LACI in patients with hypertension [22,23], aligning with our findings that systolic BP is a significant determinant of elevated LACI. Similarly, the strong correlation between LACI and triglycerides observed in our study is consistent with prior research on dyslipidemia and its role in cardiac remodeling.

While left atrial strain has been highlighted as a marker of cardiovascular health [24,25,26], LACI offers the added advantage of encompassing both atrial and ventricular mechanics, potentially bridging gaps in current risk models. The high sensitivity and specificity of LACI thresholds for predicting SCORE ≥10% and ≥5%, respectively, further reinforce its clinical relevance in identifying high-risk individuals, particularly in intermediate-risk populations.

Emerging research highlights the potential of the left atrioventricular coupling index (LACI) in evaluating cardiovascular risk in individuals with diabetes and prediabetes [27]. Recent studies have demonstrated that while patients with diabetes or prediabetes often exhibit impaired left atrial (LA) reservoir and conduit function, LACI appears to remain relatively preserved in these populations [27]. This finding suggests that LACI may provide complementary insights into cardiac function, particularly in the early stages of metabolic dysregulation, as observed in prediabetes.

Furthermore, the co-occurrence of hypertension in patients with diabetes has been shown to exacerbate alterations in atrial-ventricular dynamics, including a significant increase in LACI [23]. Notably, hypertension was independently associated with elevated LACI values and a decline in LA booster strain [23], emphasizing its role in further deteriorating atrial–ventricular coupling. These findings align with our study’s observations that systolic blood pressure significantly influences LACI, reinforcing its utility as a biomarker sensitive to hemodynamic stressors. Although these studies provide valuable insights, they also indicate that the predictive value of LACI in diabetes may vary depending on the presence of additional cardiovascular risk factors such as hypertension. The role of LACI in integrating functional and structural cardiac changes warrants further exploration, particularly in the context of early metabolic disorders where conventional markers may not fully capture cardiovascular risk.

Looking ahead, the integration of LACI into routine cardiovascular assessment may offer new opportunities to refine risk stratification, especially in patients classified as intermediate risk according to conventional scoring systems. Given its ability to reflect both structural and functional cardiac alterations, LACI could serve as a complementary imaging-based marker alongside traditional clinical and laboratory parameters. In particular, its inclusion in multiparametric models might enhance the identification of subclinical cardiac remodeling. However, before LACI can be adopted in clinical decision-making, its prognostic value, reproducibility, and incremental benefit over existing tools must be confirmed in larger, prospective cohorts. Future research should also explore whether LACI-guided risk assessment can translate into improved patient outcomes and more personalized management strategies.

Several limitations must be acknowledged. The relatively small sample size of 137 participants may limit the generalizability of our findings and reduce the statistical power to detect subtle correlations or subgroup differences. The adopted inclusion and exclusion criteria determined the nature of the study group. For example, insufficient quality of the coronary computed tomography angiography was adopted as an exclusion criterion, which resulted in the absence of patients with cardiac arrhythmias among the study participants. The study group was not representative of the general population, but rather a group of patients who needed to verify the suspicion of coronary artery disease. In the project, due to the lack of complete data on the concentrations of cholesterol fractions in blood and complete data on the concentrations of total cholesterol in blood, SCORE, not SCORE2, was used to assess cardiovascular risk, which should be considered another significant limitation of the study. Additionally, the absence of long-term outcome data prevents the assessment of LACI’s prognostic value in predicting major adverse cardiovascular events (MACEs). As such, this study does not aim to establish LACI as a predictive biomarker but rather to investigate its potential as a structural and functional marker associated with cardiovascular risk as estimated by the SCORE system. This cross-sectional design limits causal inference and clinical applicability in prognostic decision-making. Future studies should focus on validating these findings in larger, more diverse populations and exploring the integration of LACI into existing cardiovascular risk models. Longitudinal studies are also needed to determine whether LACI can predict long-term outcomes and guide therapeutic interventions.

## 6. Conclusions

LACI shows promise as a practical tool for cardiovascular risk stratification, warranting further validation in larger and more diverse populations. The findings of this study establish the left atrioventricular coupling index (LACI) as a novel and significant biomarker associated with cardiovascular risk, as reflected by its correlation with the SCORE system. These results underscore LACI’s potential to complement existing risk assessment tools, particularly in stratifying intermediate-risk groups where traditional methods may lack precision.

Key contributors to elevated LACI values, such as systolic blood pressure and triglycerides, highlight potential targets for therapeutic intervention, further supporting the clinical relevance of this biomarker. LACI’s ability to integrate structural and functional cardiac dimensions offers a unique perspective on cardiovascular health, bridging gaps in current risk models.

## Figures and Tables

**Table 1:** Basic clinical parameters in the study group (*n* = 137).

Variable	Whole Study Group
Age [years] ^b^	56.09 ± 7.64
Male sex ^a^	63/46.0
Female sex ^a^	74/54.0
BMI [kg/m^2^] ^b^	28.55 ± 5.24
Systolic blood pressure [mmHg] ^b^	140.41 ± 18.27
Diastolic blood pressure [mmHg] ^b^	84.39 ± 10.12
Total cholesterol [mg/dL] ^b^	178.13 ± 13.84
Triglicerydes [mg/dL] ^b^	97.82 ± 29.83
Smoking ^a^	38/14.1
eGFR [ml/min/1.73 m^2^] ^b^	74.45 ± 11.49
SCORE [%] ^c^	2.00 (1.00, 4.00)
SCORE < 1% ^a^	17/12.4
SCORE 1–4% ^a^	89/65.0
SCORE 5–9% ^a^	18/13.1
SCORE ≥ 10% ^a^	13/9.5

^a^ values represent absolute values/percentages; ^b^ normally distributed variable: values represent means ± standard deviation; ^c^ non-normally distributed variable: values represent median (1st quartile, 3rd quartile); BMI—body mass index; SCORE—systematic coronary risk evaluation.

**Table 2:** Basic echocardiography and coronary computed tomography angiography parameters in the study group (*n* = 137).

Variable	Whole Study Group
Echocardiography	
LVEDD [mm] ^b^	50.44 ± 5.11
LVESD [mm] ^b^	31.70 ± 4.30
IVSEDD [mm] ^b^	11.44 ± 2.46
PWEDD [mm] ^b^	10.54 ± 2.01
LA [mm] ^b^	41.46 ± 4.03
LVEF [%] ^b^	65.70 ± 5.72
E/A ^b^	1.05 ± 0.28
E/E’ ^b^	8.87 ± 2.24
Coronary computed tomography angiography	
CACS ^c^	101.00 (0.00, 268.00)
CAD-RADS 0 ^a^	60/43.8
CAD-RADS 1 ^a^	38/27.7
CAD-RADS 2 ^a^	22/16.1
CAD-RADS 3 ^a^	13/9.5
CAD-RADS 4 ^a^	3/2.2
CAD-RADS 5 ^a^	1/0.7
LVEF [%] ^b^	69.61 ± 8.37
LVM [g] ^b^	111.90 ± 31.32
LV EDV [mL] ^b^	124.89 ± 54.16
LA EDV [mL] ^b^	49.39 ± 25.39
LACI [%] ^b^	42.56 ± 21.73

^a^ values represent absolute values/percentages; ^b^ normally distributed variable: values represent means ± standard deviation; ^c^ non-normally distributed variable: values represent median (1st quartile, 3rd quartile); CACS—coronary artery calcium score; CAD—coronary artery diseases; LACI—left atrioventricular coupling index; LA EDV—left atrial end-diastolic volume; LVEF—left ventricular ejection fraction; LV EDV—left ventricular end-diastolic volume; LVM—left ventricular mass; RADS—reporting and data system.

**Table 3:** Left atrioventricular coupling index (LACI) in subgroups determined based on the SCORE questionnaire.

Grouping Variable		LACI [%] ^b^
Age [years]	≥Me (≥57)	42.55 ± 22.57
	<Me (<57)	42.57 ± 20.91
	*p*	0.48
Sex	male	51.46 ± 23.83
	female	34.98 ± 16.45
	*p*	0.01
Smoking	yes	46.71 ± 24.10
	no	41.94 ± 20.39
	*p*	0.13
Systolic blood pressure [mmHg]	≥Me (≥140)	45.52 ± 23.89
	<Me (<140)	39.56 ± 19.00
	*p*	0.04
Total cholesterol [mg/dL]	≥Me (≥175)	45.70 ± 18.71
	<Me (<175)	44.50 ± 24.20
	*p*	0.69
SCORE [%]	<1%	40.86 ± 22.62
	≥1%	42.80 ± 21.69
	*p*	0.54
	<5%	40.31 ± 20.37
	≥5%	50.27 ± 24.67
	*p*	0.02
	<10%	40.88 ± 20.26
	≥10%	58.59 ± 28.90
	*p*	0.01
CAD-RADS	<3	41.22 ± 22.46
	≥3	52.01 ± 11.18
	*p*	0.03

^b^ normally distributed variable: values represent means ± standard deviation; CAD—coronary artery diseases; LACI—left atrioventricular coupling index; Me—median; RADS—reporting and data system; SCORE—systematic coronary risk evaluation.

**Table 4:** SCORE in subgroups determined based on the left atrioventricular coupling index (LACI).

Grouping Variable		SCORE [%] ^c^	SCORE < 1% ^a^	SCORE ≥ 5% ^a^	SCORE ≥ 10% ^a^
LACI [%]	<Me (<31.84)	2.00 (1.00, 3.00)	14.7	17.6	7.4
	≥Me (≥31.84)	2.00 (1.00, 5.00)	10.1	27.5	11.6
	*p*	0.04	0.32	0.09	0.31

^a^ values represent absolute values/percentages; ^c^ non-normally distributed variable: values represent median (1st quartile, 3rd quartile); LACI—left atrioventricular coupling index; Me—median; SCORE—systematic coronary risk evaluation.

**Table 5:** Correlations between the left atrioventricular coupling index (LACI) and other quantitative variables in the study group.

	LACI [%]	
	r	*p*
Age [years]	0.08	0.43
BMI [kg/m^2^]	0.19	0.03
Systolic blood pressure [mmHg]	0.22	0.01
Diastolic blood pressure [mmHg]	0.11	0.18
Total cholesterol [mg/dL]	0.16	0.14
Triglicerydes [mg/dL]	0.44	0.01
SCORE [%]	0.29	0.01
CAD-RADS	0.13	0.28

BMI—body mass index; CAD—coronary artery diseases; LACI—left atrioventricular coupling index; RADS—reporting and data system; SCORE—systematic coronary risk evaluation.

**Table 6:** The sensitivity and specificity of left atrioventricular coupling index (LACI) as a predictor of SCORE.

	SCORE < 1%	SCORE ≥ 5%	SCORE ≥ 10%
LACI as predictor of SCORE [%]	<28.14	≥29.52	≥53.34
Sensitivity	0.767	0.462	0.798 *
Specificity	0.412	0.806 **	0.615
Accuracy	0.723	0.540	0.781 ***
Positive predictive values	0.902	0.891	0.952
Negative predictive values	0.200	0.305	0.242
Likelihood ratios positive	1.303	2.388	2.076
Likelihood ratios negative	0.567	0.667	0.328

* highest prediction sensitivity; ** highest prediction specificity; *** highest prediction accuracy; LACI—left atrioventricular coupling index; SCORE—systematic coronary risk evaluation.

## Data Availability

The datasets generated and analyzed during the current study are not publicly available but are available from the corresponding author on reasonable request.
